# A proposed method for locating the tibial attachment for medial patellotibial ligament reconstruction: A cadaveric anatomical and imaging study

**DOI:** 10.1002/jeo2.70814

**Published:** 2026-06-22

**Authors:** Xinyu Tang, Dongfang Zhang, Haitao Fu, Di Qu, Xinkun Han, Tao Li, Chao Qi

**Affiliations:** ^1^ Department of Sports Medicine, The Affiliated Hospital of Qingdao University Qingdao University Qingdao Shandong China; ^2^ Qingdao Medical College of Qingdao University Qingdao Shandong China; ^3^ Adolescent Sports Medicine Department, Qingdao Municipal Hospital University of Health and Rehabilitation Sciences Qingdao Shandong China; ^4^ Dalian Medical University Dalian Liaoning China; ^5^ Department of Joint Surgery The Affiliated Hospital of Qingdao University Shandong China

**Keywords:** anatomy, medial patellofemoral ligament, medial patellotibial ligament, patellar dislocation, tibial attachment

## Abstract

**Purpose:**

To determine the attachment sites of the medial patellofemoral ligament (MPFL) and medial patellotibial ligament (MPTL) through anatomical and imaging measurements, and to suggest a possible method for locating the tibial attachment of MPTL for reconstruction purposes.

**Methods:**

Twenty‐six adult knee specimens (16 formalin‐fixed, 10 fresh‐frozen) underwent anatomical and imaging measurements, including the morphology of MPFL and MPTL and distances from their attachment points to bony landmarks. Two parameters were used to determine the MPTL tibial attachment position: (1) Anatomical vertical distance to the tibial joint line; (2) a/b ratio on anteroposterior (AP) X‐ray. For the a/b ratio, line 1 was defined as perpendicular to the tibial joint line and passing through the tibial tuberosity centre, and line 2 was drawn parallel to line 1 and passing through the medial edge of the femoral medial epicondyle; the vertical distance from the MPTL tibial attachment to line 1 was termed ‘a’, and the distance between line 1 and line 2 was termed ‘b’.

**Results:**

The MPFL and MPTL were consistently identified in second layer of the medial retinaculum. The tibial attachment of MPTL was 14.0 ± 0.7 mm from the tibial joint line, and the a/b ratio on the AP X‐ray was 0.51 ± 0.03. Additional anatomical parameters, including ligament dimensions and distances to surrounding bony landmarks, were also documented. There were no significant differences between the results of the imaging and anatomical measurements of the distances from the ligament attachment points to the bony landmarks (*p* > 0.05).

**Conclusion:**

For MPTL reconstruction, the tibial attachment can be located 14 mm distal to the joint line and halfway between the tibial tuberosity centre and medial epicondyle (a/b = 0.51). This method serves as a reference and warrants further validation in biomechanical and reproducibility studies.

**Level of Evidence:**

Level V, cadaveric anatomical study.

**Abbreviations**
AP: anteroposterior
CI: confidence interval
MPFL: medial patellofemoral ligament
MPTL: medial patellotibial ligament
SD: standard deviation

## INTRODUCTION

The medial patellofemoral ligament (MPFL) and medial patellotibial ligament (MPTL) are important ligaments responsible for maintaining patellar stability to prevent lateral dislocation [30]. Among them, the MPFL is the primary medial stabiliser of the patella, providing 50%–60% of medial stability to the patella, and structural damage to the MPFL is found in 98.6% of acute patellar dislocations [4, 6, 7, 13, 21, 23, 24, 25, 29]. The MPTL is considered a secondary restraint, with its contribution to limiting lateral patellar displacement increasing from 26% at full knee extension to 46% at 90° of flexion [6, 24, 28]. It functions biomechanically to resist quadriceps pull in extension and, as the knee flexes, it tenses to provide substantial restraint against lateral translation, tilt and rotation [9, 11, 24].

In the treatment of lateral patellar instability, MPFL reconstruction is a common surgical procedure [3, 18, 20, 31, 34]. However, a systematic review found that 12.6% of patients still had patellar instability after surgery [27], and isolated MPFL reconstruction may not fully restore the native patellofemoral biomechanics [9], especially when performed on patients with underlying anatomical risk factors (e.g., high‐grade dysplasia with J‐sign) [35]. Preliminary clinical studies suggest that MPFL‐MPTL combined reconstruction can improve patellar stability with a low complication rate, but evidence remains limited due to small sample sizes and a lack of standardised surgical techniques [1, 2, 5, 8, 12, 22, 28]. In particular, studies on MPTL anatomy are scarce and inconsistent, and no unified anatomical or imaging reference for its tibial attachment has been established.

Therefore, the purpose of this study is to clarify the attachment sites of the MPFL and MPTL and their positional relationships with surrounding bony landmarks through anatomical and imaging studies, and to suggest a possible method for locating the tibial attachment for MPTL reconstruction based on these anatomical and imaging data. This localisation method aims to facilitate accurate tunnel placement during MPTL reconstruction and provide a foundation for future biomechanical and clinical studies.

## METHODS

### Study subjects

A total of 26 unpaired adult knee joint specimens were included in this study. These comprised 16 formalin‐fixed specimens (seven male, nine female; eight left knees, eight right knees; age range 32–65 years, mean 49 years) and 10 fresh‐frozen specimens (four male, six female; six left knees, four right knees; age range 35–53 years, mean 43 years). All 26 specimens were utilised for the anatomical and imaging studies.

All specimens were excluded for a history of knee surgery (including ligament reconstruction, meniscectomy, etc.), trauma history (such as fractures or ligament injuries), degenerative diseases (such as osteoarthritis, synovitis), developmental deformities (such as congenital high patella, trochlear dysplasia), infection history (such as septic arthritis), and other pathological changes affecting patellofemoral joint stability. All specimens included the complete knee joint structure, and the femoral and tibial shafts were osteotomized 15 cm from the joint line. In terms of experimental preparation, formalin‐fixed specimens were appropriately hydrated before dissection to restore tissue flexibility and elasticity for subsequent dissection operations. Fresh‐frozen specimens were stored in a −20°C freezer before dissection, and the specimens were immediately dissected after being thawed at room temperature for 12 h before the experiment to maintain tissue freshness.

### Research methods

#### Dissection method

**Figure 1:**
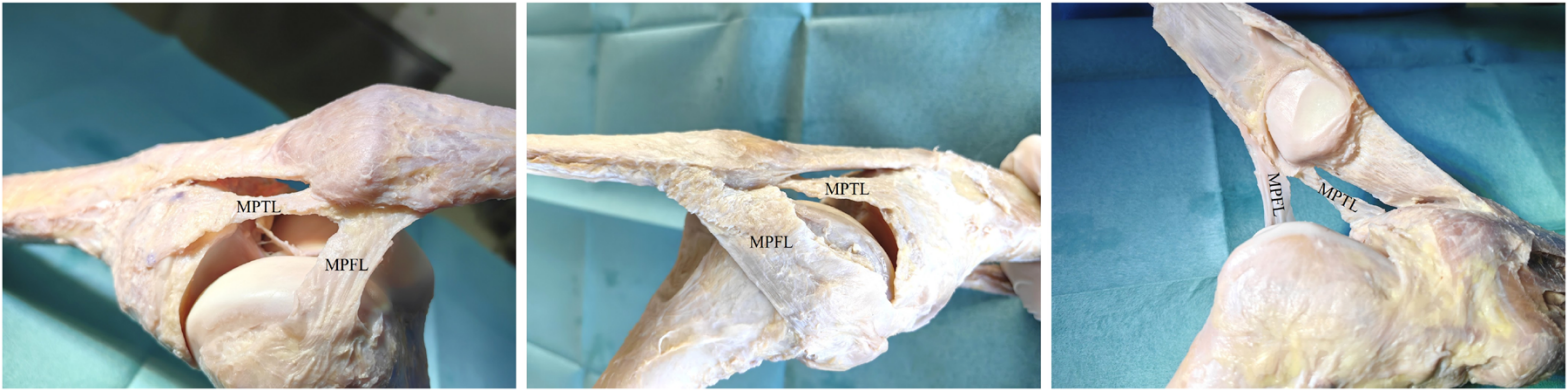
Anatomical dissection of MPFL and MPTL in formalin specimens. MPFL, medial patellofemoral ligament; MPTL, medial patellotibial ligament.

**Figure 2:**
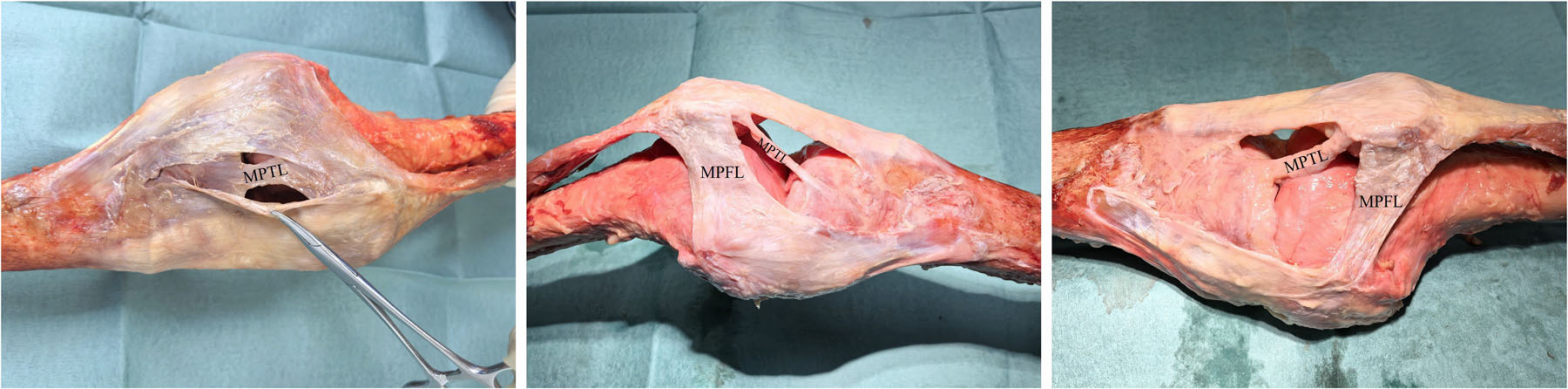
Anatomical dissection of MPFL and MPTL in fresh‐frozen specimens. MPFL, medial patellofemoral ligament; MPTL, medial patellotibial ligament.

The knee joint was fully dissected, including the sequential removal of the skin, subcutaneous tissue, and some muscles around the knee joint (including the vastus medialis, vastus lateralis, vastus intermedius, tibialis anterior, etc.) to fully expose the bony structure and ligamentous tissue of the knee joint. A parapatellar lateral dissection was performed to access the medial tissues. The ligaments were identified by combining external joint observation and surface palpation with deep palpation inside the joint. Dissection along the lower edge of the quadriceps femoris was performed to expose the upper edge of the MPFL. To dissect the MPTL from within the knee joint, a lateral knee incision was made, the infrapatellar fat pad was removed and the patella was flipped medially. The synovium and joint capsule at the anteromedial aspect of the knee joint were carefully dissected, and the MPTL was identified by exposing their attachment points on the patella and following the fibres distally to the tibial attachment. The surrounding soft tissues were removed while maintaining the integrity of the attachment points. The MPFL and MPTL were carefully separated using a blade and forceps along the direction of the ligament fibres, avoiding damage to the ligament structure and observing the fibre orientation, attachment point locations and relationships with surrounding tissues (Figures 1 and 2). All 26 specimens successfully completed the full dissection and measurement protocol, no anatomical anomalies were identified beyond the predefined exclusion criteria after dissection.

#### Measurement methods

##### Anatomical measurements

The ligament attachment points in this study were defined as the projection centres of the attachment areas of the MPFL/MPTL on the bone surface, which were determined by bisecting the proximodistal and anteroposterior dimensions of each attachment area. Anatomical measurements were performed on the dissected MPFL and MPTL using measuring tools such as calipers. The measurement content included ligament morphological parameters (length, width and thickness) and the distances from the attachment points to bony landmarks (Table 1). The measurements of the MPFL were conducted with the knee joint fully extended. The MPTL plays an important role in maintaining patellar stability at higher flexion angles [24], so its measurements were taken with the knee joint flexed at 90°. During the measurement process, the patella was manually pulled proximally and laterally to just flatten and straighten the ligament, ensuring the accuracy of the measurement results and avoiding errors introduced by rotation or twisting. The measuring tools used were digital calipers with an accuracy of 0.01 mm, and angle measurements were made using a regular goniometer with an accuracy of 1°. To ensure the reliability of the data, each parameter was measured three times, and the average of the three measurements was taken as the final data.

**Table 1:** Anatomical measurement parameters.

Ligament	Measurement category	Measurement parameters
MPFL	Morphological parameters	Ligament length, mid‐ligament thickness, patellar attachment width, femoral attachment width, mid‐ligament width
	Distances from attachment points to bony landmarks	Femoral attachment point to: femoral medial epicondyle, adductor tubercle; Patellar attachment point to: superior pole of patella, inferior pole of patella
MPTL	Morphological parameters	Ligament length, mid‐ligament thickness, patellar attachment width, tibial attachment width, mid‐ligament width, angle with patellar tendon
	Distances from attachment points to bony landmarks	Tibial attachment point to: tibial tuberosity, medial tibial condyle, tibial joint line; Patellar attachment point to: inferior pole of patella, MPFL patellar attachment point

Abbreviations: MPFL, medial patellofemoral ligament; MPTL, medial patellotibial ligament.

##### Imaging measurements

**Figure 3:**
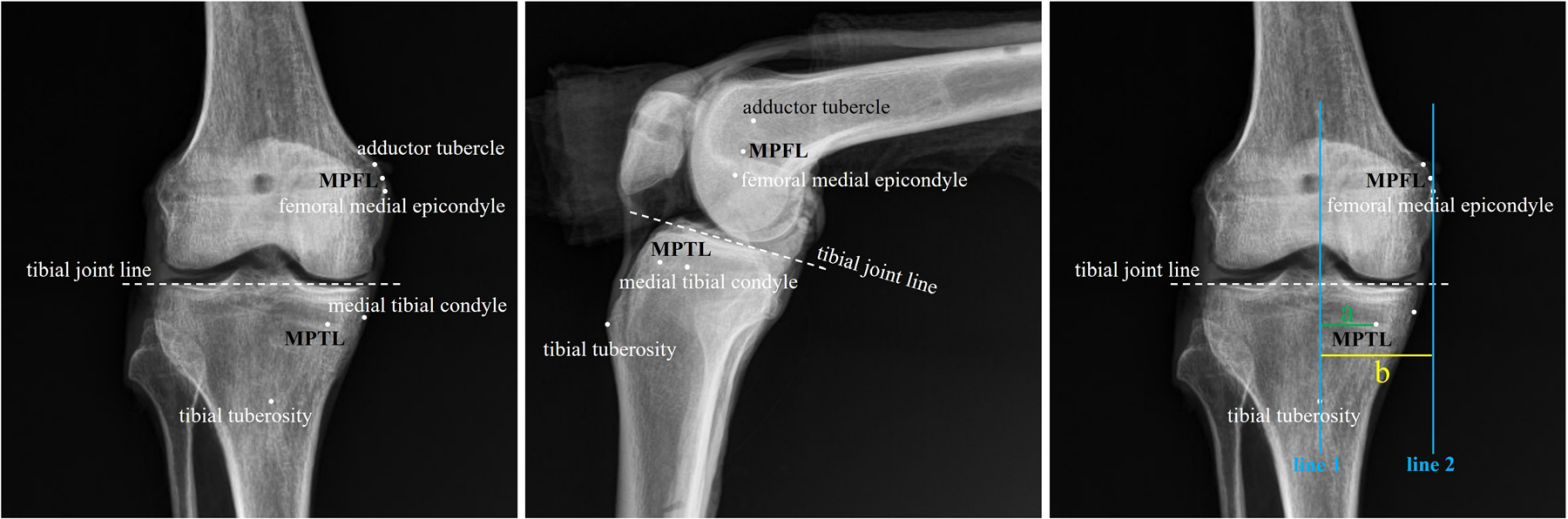
Positional relationship between the attachment sites of MPFL and MPTL and anatomical landmarks in imaging. Line 1, perpendicular to the tibial joint line, passes through the centre of the tibial tuberosity; line 2, parallel to line 1, passes through the medial edge of the femoral medial epicondyle; distance ‘a’, vertical distance from the MPTL tibial attachment point to line 1; distance ‘b’, vertical distance between line 1 and line 2; MPFL, medial patellofemoral ligament; MPTL, medial patellotibial ligament.

Metal pins were inserted into the femoral attachment point of MPFL, the tibial attachment point of MPTL, the tibial tuberosity, the medial tibial condyle, and the adductor tubercle, flush with the cortical bone surface, to ensure consistency between imaging and anatomical localisation. Subsequently, anteroposterior (AP) X‐ray films of the knee joint in full extension and lateral X‐ray films with the knee flexed at 90° were taken. On the AP view, the tibial joint line was determined by the line tangent to the proximal ends of the medial and lateral tibial plateaus. A line perpendicular to the tibial joint line and passing through the centre of the tibial tuberosity was drawn (line 1). Another line parallel to line 1 and passing through the medial edge of the femoral medial epicondyle was drawn (line 2). The vertical distance from the tibial attachment point of MPTL to line 1 (a) and the distance between line 1 and line 2 (b) were measured. On the lateral film, the tibial joint line was drawn as the tangent connecting the most superior anterior and posterior points of the medial tibial plateau (Figure 3). Specific measurement parameters are shown in Table 2.

**Table 2:** Imaging measurement parameters.

Ligament	Measurement parameters
MPFL	Distance from femoral attachment point to femoral medial epicondyle (AP and lateral views)
	Distance from femoral attachment point to adductor tubercle (AP and lateral views)
MPTL	Distance from tibial attachment point to tibial joint line (AP and lateral views)
	Distance from tibial attachment point to tibial tuberosity (AP view)
	Distance from tibial attachment point to medial tibial condyle (AP view)
	Ratio (a/b) of the vertical distance from tibial attachment point to line 1 (a) to the spacing between line 1 and line 2 (b) on the AP view

Abbreviations: AP, anteroposterior; MPFL, medial patellofemoral ligament; MPTL, medial patellotibial ligament.

### Statistical analysis

Statistical analysis was completed using SPSS 24.0 software. The normality of continuous variables was verified by the Shapiro–Wilk test. Data that met the normal distribution were expressed as mean ± standard deviation, and non‐normal data were expressed as median (interquartile range). Paired *t*‐tests were performed on paired data (such as the distances from ligament attachment points to bony landmarks), and the homogeneity of variance was confirmed by Levene's test (*p* > 0.05) before the test. The significance level was set at *p* < 0.05.

## RESULTS

### Anatomical study results

Anatomical characteristics of the MPFL: The MPFL is located in the second layer of the knee joint, originating between the femoral medial epicondyle and the adductor tubercle, and widely attaching to the medial half of the proximal patella and the medial part of the quadriceps tendon. Together with the medial femoral head of the quadriceps tendon, it forms the medial stabilising structure of the patella (Figures 1, and 2). The length of the MPFL is 47.7 ± 6.8 mm, the mid‐ligament thickness is 1.0 ± 0.2 mm, and the mid‐ligament width is 17.7 ± 2.5 mm. The patellar attachment point of the MPFL is located near the middle upper 1/3 of the medial edge of the patella. The femoral attachment point of MPFL is 8.7 ± 1.1 mm from the femoral medial epicondyle and 9.2 ± 1.5 mm from the adductor tubercle. Detailed measurement parameters are shown in Table 3.

**Table 3:** Anatomical parameters of MPFL and MPTL and distances from ligament attachment points to bony landmarks.

Measurement parameter	MPFL	MPTL
Ligament length (mm)	47.7 ± 6.8	36.8 ± 5.5
Mid‐ligament thickness (mm)	1.0 ± 0.2	1.2 ± 0.3
Patellar attachment width (mm)	22.6 ± 3.6	9.7 ± 1.4
Femoral/Tibial attachment width (mm)	18.3 ± 3.1 (Femoral)	10.9 ± 1.8 (Tibial)
Mid‐ligament width (mm)	17.7 ± 2.5	7.8 ± 1.5
Femoral attachment point to femoral medial epicondyle (mm)	8.7 ± 1.1	—
Femoral attachment point to adductor tubercle (mm)	9.2 ± 1.5	—
Tibial attachment point to tibial tuberosity (mm)	—	46.7 ± 5.2
Tibial attachment point to medial tibial condyle (mm)	—	42.9 ± 4.7
Tibial attachment point to tibial joint line (mm)	—	14.0 ± 0.7
Patellar attachment point to superior pole of patella (mm)	30.0 ± 4.6	—
Patellar attachment point to inferior pole of patella (mm)	37.5 ± 5.6	21.6 ± 3.7
Angle with patellar tendon (°)	—	19.2 ± 4.2

*Note*: Results are presented as mean ± SD.

Abbreviations: MPFL, medial patellofemoral ligament; MPTL, medial patellotibial ligament; SD, standard deviation.

Anatomical characteristics of the MPTL: The MPTL originates from the middle lower part of the medial edge of the patella, with its fibres extending downward to the anterior area of the medial tibial plateau. It is located in the second layer of the medial knee support band, adjacent to the joint capsule and the anterior horn of the medial meniscus. In some specimens, the MPTL is wrapped by the infrapatellar fat, with its patellar attachment point blending into the deep fibrous support band and some fibres closely connected to the joint capsule. Its tibial attachment is close to the medial side of the patellar tendon (Figures 1 and 2). The length of the MPTL is 36.8 ± 5.5 mm, the mid‐ligament thickness is 1.2 ± 0.3 mm, and the mid‐ligament width is 7.8 ± 1.5 mm. The tibial attachment point of MPTL is 14.0 ± 0.7 mm from the tibial joint line, 46.7 ± 5.2 mm from the tibial tuberosity, and 42.9 ± 4.7 mm from the medial tibial condyle. The distance between the patellar attachment points of the MPTL and MPFL is 20.8 ± 2.6 mm. The angle between MPTL and patellar tendon is 19.2 ± 4.2°. Detailed measurement parameters are shown in Table 3.

### Imaging study results

AP view: The femoral attachment point of MPFL is 7.4 ± 0.8 mm from the femoral medial epicondyle and 6.3 ± 0.5 mm from the adductor tubercle. The tibial attachment point of MPTL is 45.3 ± 4.7 mm from the tibial tuberosity, 41.0 ± 4.2 mm from the medial tibial condyle, and 13.7 ± 0.7 mm from the tibial joint line. The ratio (a/b) of the vertical distance from the tibial attachment point to line 1 (a) to the spacing between line 1 and line 2 (b) is 0.51 ± 0.03 (Table 4, Figure 3).

**Table 4:** Imaging measurement results of ligament attachment points‐bony landmark distances.

Measurement parameter	AP film	Lateral film
MPFL		
Distance from femoral attachment point to femoral medial epicondyle (mm)	7.4 ± 0.8	8.4 ± 1.2
Distance from femoral attachment point to adductor tubercle (mm)	6.3 ± 0.5	8.6 ± 1.1
MPTL		
Distance from tibial attachment point to tibial tuberosity (mm)	45.3 ± 4.7	—
Distance from tibial attachment point to medial tibial condyle (mm)	41.0 ± 4.2	—
Distance from tibial attachment point to tibial joint line (mm)	13.7 ± 0.7	13.5 ± 0.6
Ratio of the vertical distance from the tibial attachment point to line 1 to the spacing between line 1 and line 2 (a/b)	0.51 ± 0.03	—

*Note*: Results are presented as mean ± SD.

Abbreviations: AP, anteroposterior; MPFL, medial patellofemoral ligament; MPTL, medial patellotibial ligament; SD, standard deviation.

Lateral view: The femoral attachment point of MPFL is 8.4 ± 1.2 mm from the femoral medial epicondyle and 8.6 ± 1.1 mm from the adductor tubercle. The tibial attachment point of MPTL is 13.5 ± 0.6 mm from the tibial joint line (Table 4, Figure 3).

There were no significant differences between the results of the imaging and anatomical measurements of the distances from the ligament attachment points to the bony landmarks (*p* > 0.05) (Table 5). Based on the anatomical and imaging studies of the MPTL, the anatomical distance from the tibial attachment point of MPTL to the tibial joint line is 14.0 ± 0.7 mm, and the a/b ratio on the AP X‐ray is 0.51 ± 0.03.

**Table 5:** Comparison of imaging and anatomical measurement results (mm).

Measurement parameter	Imaging	Anatomy	*p*
MPFL (Lateral film)			
Distance from femoral attachment point to femoral medial epicondyle	8.4 ± 1.2	8.7 ± 1.1	n.s.
Distance from femoral attachment point to adductor tubercle	8.6 ± 1.1	9.2 ± 1.5	n.s.
MPTL (AP film)			
Distance from tibial attachment point to tibial tuberosity	45.3 ± 4.7	46.7 ± 5.2	n.s.
Distance from tibial attachment point to medial tibial condyle	41.0 ± 4.2	42.9 ± 4.7	n.s.
Distance from tibial attachment point to tibial joint line	13.7 ± 0.7	14.0 ± 0.7	n.s.

*Note*: Results are presented as mean ± SD, n.s. indicates *p* > 0.05.

Abbreviations: AP, anteroposterior; MPFL, medial patellofemoral ligament; MPTL, medial patellotibial ligament; SD, standard deviation.

## DISCUSSION

The most important finding of this study is the proposal of a method for locating the tibial attachment point for MPTL reconstruction based on anatomical and imaging studies. Through anatomical and imaging studies on formalin‐fixed and fresh‐frozen knee joint specimens, this study focused on quantifying the positional relationship between the tibial attachment point of MPTL and key bony landmarks, and proposed the anatomical distance of 14.0 ± 0.7 mm from the tibial joint line, combined with the a/b ratio on the AP X‐ray (0.51 ± 0.03) as an objective reference indicator for locating the tibial attachment point for MPTL reconstruction. These findings provide anatomical and imaging basis for the application of MPFL‐MPTL combined reconstruction strategies in clinical practice.

Compared with the extensive research on the MPFL, the anatomical characteristics and positioning standards of the MPTL have been relatively less studied and are inconsistent [10, 14, 16]. Our anatomical study confirmed its location in the second layer of the medial retinaculum, which is consistent with some previous studies [15, 18, 33]. Although our quantitative measurements of the MPTL (Table 3) generally fall within the range reported in the literature [15, 17, 18, 19], there are some differences in certain parameters (such as the distance from the tibial attachment of MPTL to the tibial tuberosity and the angle between the MPTL and the patellar tendon) compared with some studies [10, 19, 25]. This observed heterogeneity underscores the limitations of relying solely on fixed distances from bony landmarks and highlights the need for a more standardised and reproducible localisation method.

The position of the MPTL tibial attachment determines its synergistic angle with the MPFL. Because bone size varies among patients, a ratio‐based parameter may be more generalisable than absolute distances or angles when evaluating the tibial site for MPTL reconstruction. Therefore, this study defined two anatomical reference lines on the AP view (Figure 3) and measured the a/b ratio, which was 0.51 ± 0.03. Combined with anatomical data (the attachment point is located 14.0 ± 0.7 mm distal to the tibial joint line), this means that the tibial site for MPTL reconstruction can be selected approximately halfway between the centre of the tibial tuberosity and the medial edge of the femoral medial epicondyle and 14 mm distal to the tibial joint line.

Although imaging does not fully cover anatomical parameters, the measured ligament attachment point‐bony landmark distances and a/b ratios are the core parameters for intraoperative fluoroscopic navigation. During clinical surgery, the original ligament attachment points are not directly visible unless intraoperative exposure is performed; therefore, it is necessary to rely on anatomical and imaging parameters to assess the ligament attachment areas.

There are some limitations to this study. First, the sample size limits subgroup analysis of the population. Second, the study used formalin‐fixed and fresh‐frozen specimens, which may differ from in vivo conditions, particularly without considering the effects of dynamic factors in living subjects (such as quadriceps contraction and patellar tendon tension changes) on ligament tension. Third, no a priori sample size calculation was performed. However, post hoc precision analysis confirmed that the sample size (*n* = 26) provided stable estimates for the key parameters, and our sample size was comparable to or larger than those in previous anatomical studies of the MPFL or MPTL [15, 18, 25, 26, 32]. Future studies could based on the MPTL tibial attachment localisation method established in this study to conduct biomechanical investigations of MPFL‐MPTL combined reconstruction, and should include reproducibility testing of this method and larger sample sizes for further validation.

## CONCLUSION

For MPTL reconstruction, the tibial attachment can be located 14 mm distal to the joint line and halfway between the tibial tuberosity centre and medial epicondyle (a/b = 0.51). Future biomechanical and reproducibility studies are warranted to validate the efficacy of this approach in improving patellar stability.

## AUTHOR CONTRIBUTIONS

**Xinyu Tang**: Conceptualisation; methodology; formal analysis; investigation; writing—original draft. **Dongfang Zhang**: Methodology; formal analysis; investigation; writing—original draft. **Haitao Fu**: Methodology; investigation; funding acquisition. **Di Qu**: Methodology; investigation. **Xinkun Han**: Formal analysis; investigation. **Tao Li**: Methodology; visualisation; writing—review and editing. **Chao Qi**: Conceptualisation; methodology; resources; supervision; project administration; writing—review and editing.

## FUNDING

National Natural Science Foundation of China, Grant/Award Number: 82102558.

## CONFLICT OF INTEREST STATEMENT

The authors declare no conflicts of interest.

## ETHICS STATEMENT

The study protocol was reviewed and approved by the Medical Ethics Committee of the Affiliated Hospital of Qingdao University (QYFY WZLL 30514). All knee joint specimens used in this study were sourced from the body donation programs of Qingdao University and its affiliated hospital, and were exclusively used for educational and research purposes. After the completion of the study, all used specimens were recovered and processed in accordance with standard operating procedures to ensure compliance with ethical requirements. Information of all specimen donors was strictly kept confidential.

## Data Availability

The data within this paper are available from the corresponding author upon reasonable request.
